# ﻿Three new species of dragon pseudoscorpions (Pseudoscorpiones, Pseudotyrannochthoniidae) from China

**DOI:** 10.3897/zookeys.1204.111842

**Published:** 2024-06-04

**Authors:** Yanmeng Hou, Feng Zhang

**Affiliations:** 1 College of Life Sciences, Capital Normal University, 105 Xisanhuanbeilu, Haidian District, Beijing 100048, China Hebei University Baoding China; 2 The Key Laboratory of Zoological Systematics and Application, Institute of Life Science and Green Development, College of Life sciences, Hebei University, Baoding, Hebei 071002, China Capital Normal University Beijing China

**Keywords:** *Allochthonius*, morphology, *Spelaeochthonius*, taxonomy

## Abstract

Three new pseudoscorpions in the family Pseudotyrannochthoniidae are described from China: *Allochthoniushispidus***sp. nov.** from Chongqing (Wushan County), *Spelaeochthoniushuanglaoensis***sp. nov.** from Beijing (Fangshan District), and *Spelaeochthoniustuoliangensis***sp. nov.** from Hebei (Pingshan County). Detailed diagnoses and illustrations of all new species are provided.

## ﻿Introduction

**Figure 1.**
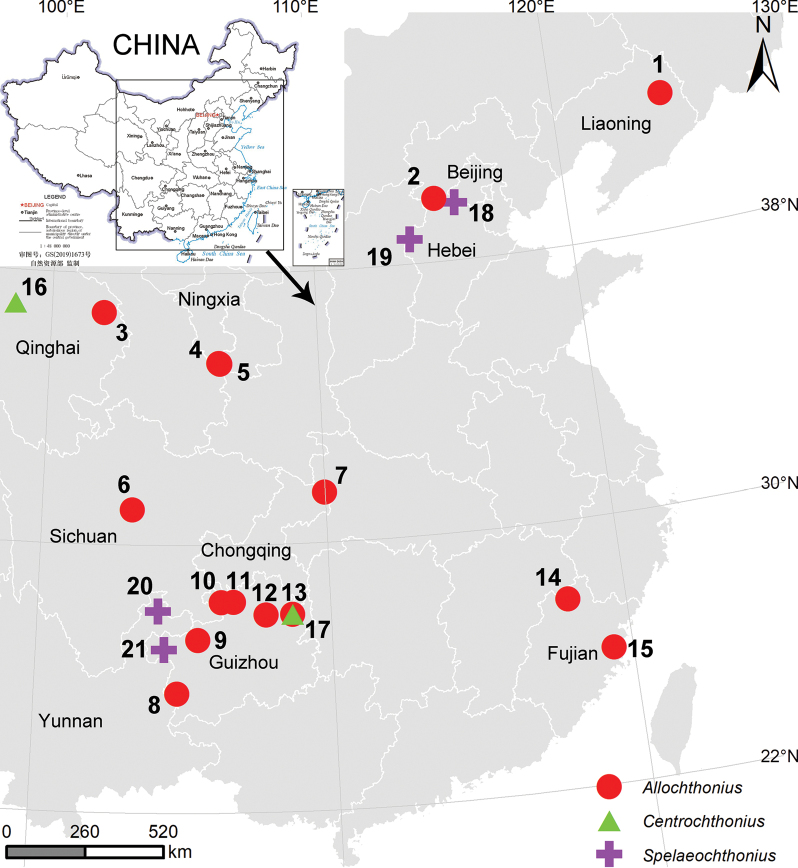
Distribution of all Pseudotyrannochthoniidae species in China. **1***Allochthoniusliaoningensis* Hu & Zhang, 2012 **2***A.exornatus* Gao & Zhang, 2013 **3***A.wui* Hu & Zhang, 2011 **4***A.jingyuanus* Zhang & Zhang, 2014 **5***A.brevitus***6***A.sichuanensis* (Schawaller, 1995) **7***A.hispidus* sp. nov. **8***A.lini* Li, 2023 **9***A.xuae* Li, 2023 **10***A.bainiensis***11***A.pandus***12***A.xinqiaoensis***13***A.fanjingshan* Gao, Zhang & Zhang, 2016 **14***A.trigonus* Hu & Zhang, 2011 **15***A.fuscus* Hu & Zhang, 2011 **16***Centrochthoniuskozlovi* (Redikorzev, 1918) **17***C.cheni* (Gao, Zhang & Zhang, 2016) **18***Spelaeochthoniushuanglaoensis* sp. nov. **19***S.tuoliangensis* sp. nov. **20***S.yinae***21***S.wulibeiensis*.

The monophyletic pseudoscorpion family Pseudotyrannochthoniidae Beier, 1932, originated in East Asia during the Middle Triassic (Harms et al. 2024) and is one of the earliest branches of pseudoscorpion families. The group is small-bodied, usually less than 3 mm, but the chelicerae are disproportionately large and resemble the jaws of the mythical dragon. Consequently, its group has earned the colloquial name dragon pseudoscorpions (Harms et al. 2024). Members of the family can be distinguished from all other pseudoscorpions in having trichobothria *ib* and *isb* located at the base of the fixed chelal finger and coxal spines present only on coxae I (Harms and Harvey 2013). Pseudotyrannochthoniidae are distributed on all continents except Antarctica and inhabit leaf litter and caves (You et al. 2022; Gao et al. 2023). Niche modeling suggests that the distribution of pseudotyrannochthoniids is determined by the interaction of constantly moderate temperatures and high moisture availability, a pattern that is globally repeated (Harms 2018; Harms et al. 2019). To date, this group comprises 80 described species in six genera. Throughout Asia, pseudotyrannochthoniids are represented by three genera, *Allochthonius* Chamberlin, 1929, *Centrochthonius* Beier, 1931, and *Spelaeochthonius* Morikawa, 1954, and all extant species in these three genera are narrow-range endemics (Fig. 1; WPC 2024).

The monophyly of both *Allochthonius* and *Spelaeochthonius* receives high support (Harms et al. 2024). The genus *Allochthonius* comprises 34 species, with 14 species documented from China and the remainder distributed across Russia, Japan, and South Korea. It is diagnosed by the carapace frequently having 22–28 setae (but fewer in some cave-dwelling congeners; Sakayori 2000; Viana and Ferreira 2021; Gao et al. 2023), coxal spines present on a common protuberance, spray- or fan-shaped, and the intercoxal tubercle generally larger (Harvey and Harms 2022; Schwarze et al. 2022). About 35% (12 of 34) of the species in this genus lack eyes, with almost all of them being cave-dwelling, except for *A.brevitus* Hu & Zhang, 2012, which is the only epigean species (Morikawa 1954, 1956, 1960; Hu and Zhang 2012; Zhang and Zhang 2014; Viana and Ferreira 2021; Gao et al. 2023).

The genus *Spelaeochthonius*, currently found only in East Asia, includes 11 described species. It can be distinguished from other pseudotyrannochthoniid genera by the number of carapaceal setae (only 16), the number, shape, and arrangement of the coxal spines (never on a common protuberance and more than seven blades that are longer and distally pinnate or serrate), and the shape of the intercoxal tubercle (bisetose and generally smaller than that of *Allochthonius*) (Morikawa 1956; You et al. 2022). The genus consists exclusively of subterranean species with strongly troglobitic habitus occurring in China (two species), South Korea (three species), and Japan (six species) (WPC 2024).

This study describes three new pseudotyrannochthoniid species from both the surface and subterranean environments. Detailed diagnoses, descriptions, and illustrations are provided for each species. Two of these species are placed in *Spelaeochthonius*, while one is assigned to *Allochthonius*. Additionally, a distribution map of all Chinese pseudotyrannochthoniid species is given.

## ﻿Materials and methods

The specimens examined for this study are preserved in 75% alcohol and deposited in the Museum of Hebei University (**MHBU**), Baoding, China, and the Museum of Southwest University (**MSWU**), Chongqing, China. Photographs, drawings, and measurements were taken using a Leica M205A stereomicroscope equipped with a Leica DFC550 camera. Detailed examination was carried out under an Olympus BX53 upright microscope. Scanning electron microscopy (SEM) was done under high vacuum with a JEOL JSM-IT500 after critical-point drying and gold-palladium coating. The distribution map was made using ArcGIS v. 10.6 (Fig. 1). All images were edited and formatted using Inkscape v. 1.0.2.0 and Adobe Photoshop 2022.

Terminology and measurements follow Chamberlin (1931) with minor modifications to the terminology of trichobothria (Harvey 1992; Judson 2007) and chelicera (Judson 2007). The chela and legs were measured in lateral view and others were taken in dorsal view. All measurements are given in mm unless noted otherwise. Proportions and measurements of chelicerae, carapace and pedipalps correspond to length/breadth, and those of legs to length/depth. For abbreviations of trichobothria, see Chamberlin (1931).

## ﻿Taxonomy

**Family Pseudotyrannochthoniidae Beier, 1932**

### ﻿Genus Allochthonius

Taxon classificationAnimaliaPseudoscorpionesPseudotyrannochthoniidae

Chamberlin, 1929

64EFF0D8-7DF0-505D-942B-DFA415E21766

#### Type species.

*Chthoniusopticus* Ellingsen, 1907, by original designation.

### ﻿ Allochthonius hispidus sp. nov.

Taxon classificationAnimaliaPseudoscorpionesPseudotyrannochthoniidae

23FA9053-9576-5394-B446-3D02571EC43F

https://zoobank.org/FB289038-20FB-46BD-8B36-07A04E1BF95B

Figs 1 , 2 , 3 , 4

#### Chinese name.

多毛异伪蝎.

#### Type materials.

***Holotype***: China • ♂; Chongqing Municipality, Wushan County, Dangyang Town, Wushanya; 31°28.356′N, 109°59.172′E; 1740 m a.s.l.; 02 Oct. 2021; Luyu Wang leg. (Fig. 1); Ps.-MHBU-CQWLP-21-02-01. ***Paratypes***: • 2♂1♀; same data as for holotype; Ps.-MHBU-CQWLP-21-02-02–04 • 2♂1♀; Wushan County, Dangyang Town, Qizhi Mountain; 31°28.109′N, 109°58.716′E; 1475 m a.s.l.; same collector and collection date as for holotype; Ps.-MHBU-CQWLP-21-03-01–03 • 1♂1♀; Wushan County, Dangyang Town, Congping Mountain; 31°23.786′N, 110°2.467′E; 2150 m a.s.l.; 03 Oct. 2021; same collector as for holotype; Ps.-MHBU-CQWLP-21-07-01 & 02• 1♀; Wushan County, Dangyang Town, Congping Management Station; 31°23.786′N, 110°2.055′E; 1970 m a.s.l.; 03 Oct. 2021; same collector as for holotype; Ps.-MHBU-CQWLP-21-08-01• 1♂1♀; Wushan County, Guanyang Town, Pingqian Management Station; 31°22.379′N, 109°56.287′E; 1832 m a.s.l.; 04 Oct. 2021; same collector as for holotype; Ps.-MSWU-CQWLP-21-10-01 & 02.

#### Diagnosis

**(♂♀).***Allochthoniushispidus* sp. nov. is most similar to another epigean blind species from China, *A.brevitus*, but differs from this species in having more carapaceal setae (22–24 (♂), 21 or 22 (♀) for *A.hispidus* vs 16 (♂♀) for *A.brevitus*), more cheliceral setae (♂) (10 or 11 vs seven), more numerous chelal fingers teeth (♂) (fixed finger with 26–29 vs 18–20 teeth, movable finger with 22 or 23 vs 17 or 18 teeth), and longer pedipalps (e.g. palpal femur 5.19 (♂), 5.13–5.61 (♀) × vs 4.33–4.73 (♂), 4.79–4.92 (♀) × longer than broad, length 1.09 (♂), 1.18–1.29 (♀) mm vs 0.52–0.57 (♂), 0.64–0.67 (♀) mm; chela length 1.59–1.60 (♂), 1.76–1.84 (♀) mm vs 0.80–0.84 (♂), 0.98–1.01 (♀) mm). It differs from the other blind species in China (*A.bainiensis* Gao, Hou & Zhang, 2023, *A.pandus* Gao, Hou & Zhang, 2023, and *A.xinqiaoensis* Gao, Hou & Zhang, 2023) in having more numerous carapaceal setae (♀) (the latter three with only 14 setae) and the presence of a pair of hirsute pedipalps. It also differs from all blind congeners from Japan (*A.yoshizawai* Viana & Ferreira, 2021, *A.ishikawai* Morikawa, 1954, and its subspecies) in having more cheliceral setae (♂) (10 or 11 vs at most seven) and more numerous fixed chelal finger teeth (♂) (26–29 vs at most 17).

#### Etymology.

The specific name is derived from the Latin word *hispidus* (hirsute, hairy), which refers to the presence of abundant setae on the chela, palpal femur, and patella.

#### Description.

**Figure 2.**
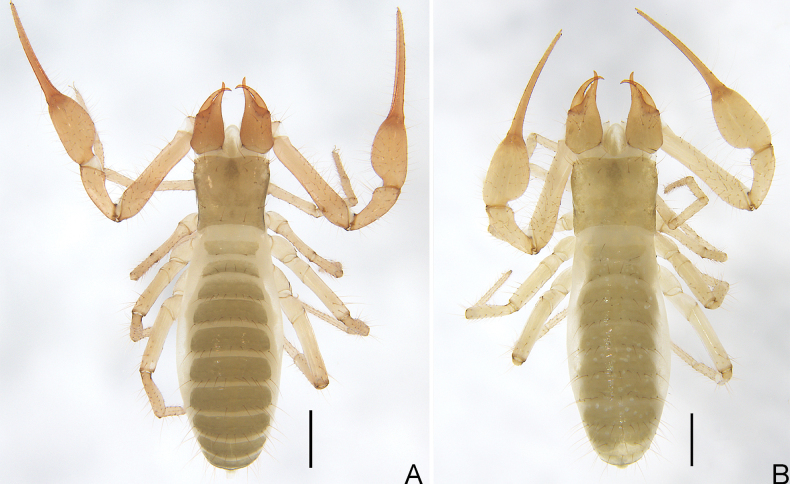
*Allochthoniushispidus* sp. nov. **A** holotype male, habitus (dorsal view) **B** paratype female, habitus (dorsal view). Scale bars: 0.50 mm.

**Figure 3.**
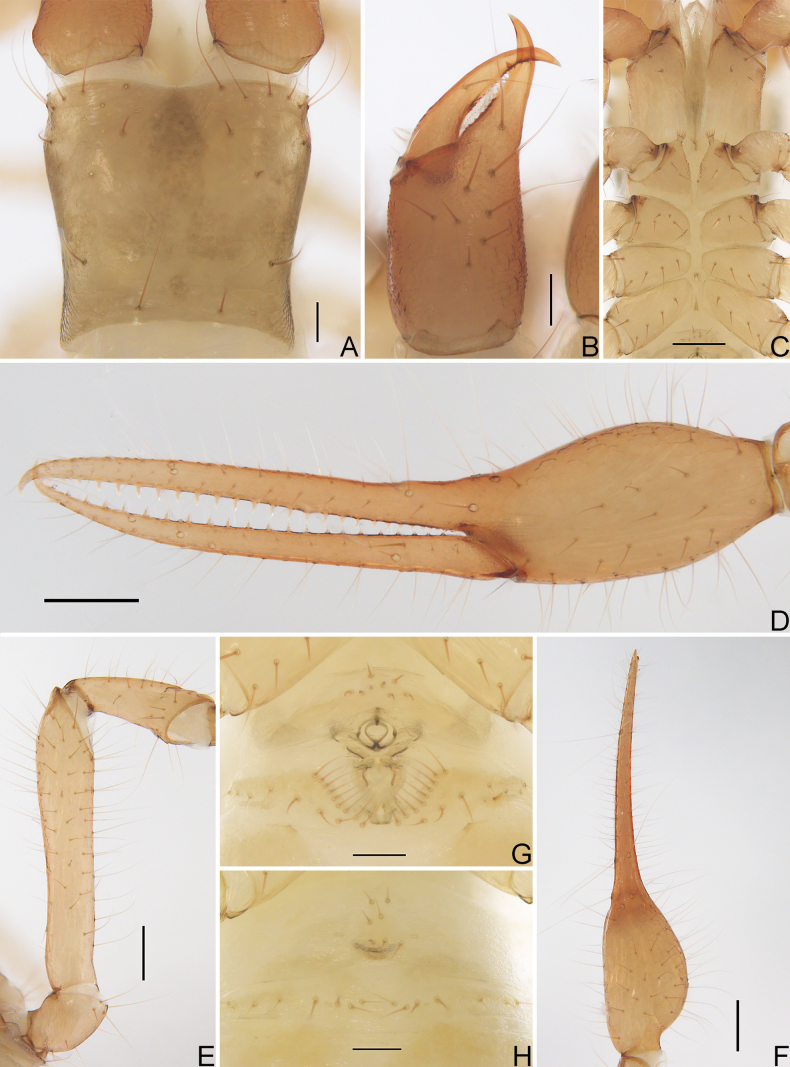
*Allochthoniushispidus* sp. nov. **A** carapace (dorsal view) **B** left chelicera (dorsal view) **C** coxae (ventral view) **D** left chela (lateral view) **E** left pedipalp (minus chela, dorsal view) **F** left chela (dorsal view) **G** male genital area (ventral view) **H** female genital area (ventral view). Scale bars: 0.20 mm (**C–F**); 0.10 mm (**A, B, G, H**).

**Figure 4.**
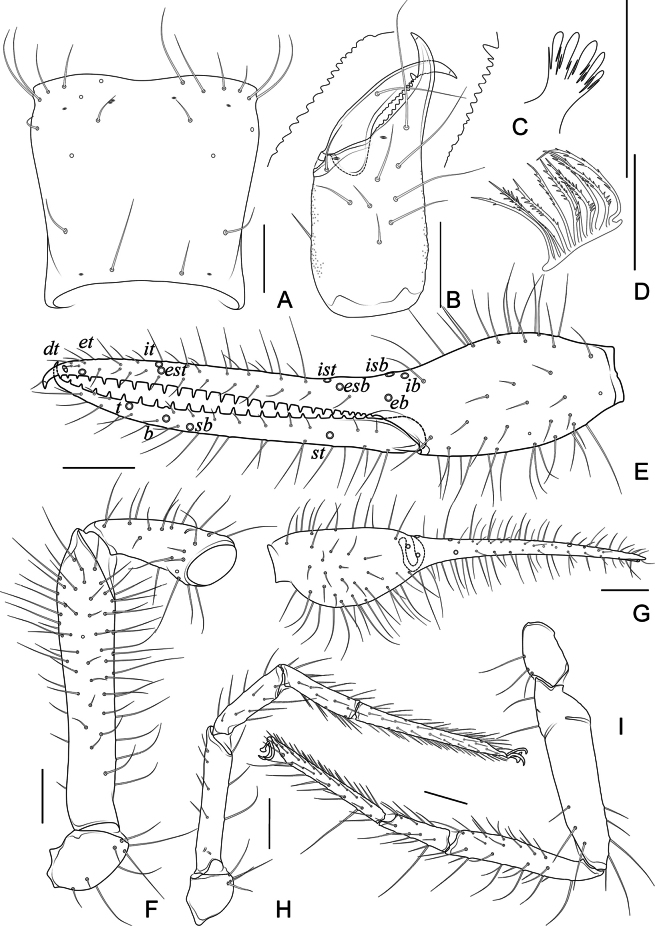
*Allochthoniushispidus* sp. nov., holotype male **A** carapace (dorsal view) **B** left chelicera (dorsal view), with details of teeth **C** coxal spines on coxae I (ventral view) **D** rallum **E** left chela (lateral view), with details of trichobothrial pattern **F** left pedipalp (minus chela, dorsal view) **G** left chela (dorsal view) **H** leg I (lateral view) **I** leg IV (lateral view). Scale bars: 0.20 mm.

**Adult males** (Figs 1, 2A, 3A–G, 4). ***Colour***: generally pale yellow; chelicerae, pedipalps and tergites slightly darker; soft parts pale. ***Cephalothorax*** (Figs 3A, C, 4A, C): carapace subquadrate, 0.87–0.88× longer than broad, gently narrowed posteriorly; surface smooth but the posterior lateral parts with squamous sculpturing; without furrows but with five anterior lyrifissures and two posterior lyrifissures; no traces of eyes; epistome absent, space between median setae slightly recurved; with 22–24 setae arranged 12–14: 4: 2: 2: 2, most setae heavy, long and gently curved. Chaetotaxy of coxae: P 3, I 3–4, II 5–6, III 4–5, IV 5; manducatory process with two acuminate distal setae, anterior seta more than 1/2 length of medial seta; coxal spines present on coxa I only, consisting of a tubercle expanded terminally into a characteristic spray- or fan-shaped of five elevate processes which extend apically, subequal in length (Figs 3C, 4C); a larger bisetose intercoxal tubercle present between coxae III and IV (Fig. 3C). ***Chelicera*** (Figs 3B, 4B, D): large, approximately as long as carapace, 2.52× longer than broad; nine or 10 setae and two lyrifissures (exterior condylar lyrifissure and exterior lyrifissure) present on hand, all setae acuminate, ventrobasal seta shorter than others; movable finger with one medial seta. Cheliceral palm with moderate hispid granulation on both ventral and dorsal sides. Both fingers with well-developed teeth, fixed finger with eight or nine acute teeth, distal one largest, plus five or six small basal teeth, 13–15 in total; movable finger with 15 or 16 retrorse contiguous teeth of equal length; galea absent. Serrula exterior with 20 or 21 blades and serrula interior with 12–14 blades. Rallum in two rows and composed of 11 finely pinnate blades (Fig. 4D). ***Pedipalp*** (Figs 3D–F, 4E–G): long and slender, trochanter 1.38–1.48, femur 5.19, patella 2.62–2.89, chela 4.82–5.16, hand 1.79–1.90× longer than broad; femur 1.98× longer than patella; movable chelal finger 1.73–1.76× longer than hand and 0.64–0.65× longer than chela. Setae generally long and acuminate; one distal lyrifissure present on patella (Figs 3E, 4F). Chelal palm robust and slightly constricted towards fingers. Fixed chelal finger and hand with eight trichobothria plus duplex trichobothrium (*dt*), movable chelal finger with four trichobothria, *ib*, *isb*, *eb*, *esb*, and *ist* clustered at the base of fixed finger, *ist* slightly distal to *esb*; *it* slightly distal to *est*, situated subdistally; *et* situated subdistally, very close to chelal teeth; *dt* situated distal to *et*, near the tip of fixed finger; *sb* situated closer to *b* than to *st* (Fig. 4E). Abundant setae present on palpal femur, patella, and chela. Sensilla absent. Both chelal fingers with a row of teeth, homodentate, spaced regularly along the margin, larger and well-spaced teeth present in the middle of the row, becoming smaller and closer distally and proximally: fixed chelal finger with 26–29 teeth, slightly retrorse and pointed; movable chelal finger with 22 or 23 teeth (slightly smaller than teeth on fixed chelal finger) and a tubercle between the seventh and eighth teeth (Figs 3D, 4E). Chelal fingers slightly curved in dorsal view (Figs 3F, 4G). ***Opisthosoma***: generally typical, pleural membrane finely granulated. Tergites and sternites undivided; setae uniseriate and acuminate. Tergal chaetotaxy I–XII: 2: 6–8: 8–10: 10–11: 10–11: 11–12: 10–13: 13–14: 8: 6: TT: 0. Sternal chaetotaxy III–XII: 12–14: 15–17: 14–15: 12–15: 12–14: 13–14: 12: 8–9: 0: 2. Anterior genital operculum with eight or nine setae, genital opening pit-like, with seven or 10 marginal setae on each side, 26 in total, with a pair of lyrifissures present anterolateral and posterolateral to genital opening, respectively (Fig. 3G). ***Legs*** (Fig. 4H, I): generally typical, long, and slender. Fine granulation present on anterodorsal faces of femur IV and patella IV. Femur of leg I 1.45× longer than patella and with two lyrifissures at the base of femur; tarsus 2.00× longer than tibia. Femoropatella of leg IV 3.48–3.70× longer than deep and with one lyrifissure at the base of femur; tibia 5.25–5.67× longer than deep; with basal tactile setae on both tarsal segments: basitarsus 3.44–3.56× longer than deep (TS = 0.25–0.32), telotarsus 8.86–9.14× longer than deep and 2.00× longer than basitarsus (TS = 0.17–0.21). Arolium slightly shorter than the claws, not divided; claws simple. ***Dimensions of adult males*** (length/breadth or, in the case of the legs, length/depth in mm): body length 2.72–2.78. Pedipalps: trochanter 0.29–0.31/0.21, femur 1.09/0.21, patella 0.55/0.19–0.21, chela 1.59–1.60/0.31–0.33, hand 0.59/0.31–0.33, movable finger length 1.02–1.04. Chelicera 0.68/0.27, movable finger length 0.36–0.38. Carapace 0.56–0.58/0.64–0.67. Leg I: trochanter 0.19–0.20/0.17–0.18, femur 0.55/0.11, patella 0.38/0.09–0.10, tibia 0.31–0.32/0.08, tarsus 0.62–0.64/0.06. Leg IV: trochanter 0.31–0.32/0.18–0.19, femoropatella 0.80–0.85/0.23, tibia 0.63–0.68/0.12, basitarsus 0.31–0.32/0.09, telotarsus 0.62–0.64/0.07.

**Adult females** (Figs 2B, 3H). Mostly same as males but a little larger (i.e. body length is about 1.08× that of males); cheliceral hand of one female with 11 setae; chaetotaxy of coxae: P 3, I 4, II 4–5, III 5, IV 5; tergal chaetotaxy I–XII: 2: 6: 6–8: 10: 10–11: 11–12: 12: 11–12: 8–9: 5: TT: 0; sternal chaetotaxy IV–XII: 15–17: 11–13: 13–14: 13–14: 13: 12–14: 9: 0: 2; anterior genital operculum with eight setae, posterior margin with 15 or 17 marginal setae, 23–25 in total; leg IV with a long tactile seta on both tarsal segments: basitarsus 3.33–3.50× longer than deep (TS = 0.37–0.40), telotarsus 9.57–11.00× longer than deep and 1.91–2.20× longer than basitarsus (TS = 0.20–0.21). Body length 2.44–2.97. Pedipalps: trochanter 0.32–0.37/0.22–0.24 (1.33–1.68×), femur 1.18–1.29/0.23 (5.13–5.61×), patella 0.59–0.60/0.22–0.24 (2.46–2.73×), chela 1.76–1.84/0.34–0.41 (4.49–5.18×), hand 0.64–0.68/0.34–0.41 (1.66–1.88×), movable chelal finger length 1.15–1.20. Chelicera 0.77–0.81/0.32–0.36 (2.25–2.41×), movable finger length 0.42–0.46. Carapace 0.58–0.64/0.72–0.83 (0.77–0.81×). Leg I: trochanter 0.23/0.19 (1.21×), femur 0.56–0.61/0.11–0.12 (4.67–5.55×), patella 0.38–0.39/0.10–0.11 (3.55–3.80×), tibia 0.35/0.07–0.08 (4.38–5.00×), tarsus 0.68–0.69/0.06–0.07 (9.71–11.50×). Leg IV: trochanter 0.33–0.35/0.20 (1.65–1.75×), femoropatella 0.85–0.90/0.25 (3.40–3.60×), tibia 0.68/0.13–0.14 (4.86–5.23×), basitarsus 0.30–0.35/0.09–0.10 (3.33–3.50×), telotarsus 0.66–0.67/0.06–0.07 (9.57–11.00×).

#### Distribution.

China (Chongqing).

### ﻿Genus Spelaeochthonius

Taxon classificationAnimaliaPseudoscorpionesPseudotyrannochthoniidae

Morikawa, 1954

B41899B6-70A2-5760-8EEE-769892380ADE

#### Type species.

*Spelaeochthoniuskubotai* Morikawa, 1954, by original designation.

### ﻿ Spelaeochthonius huanglaoensis sp. nov.

Taxon classificationAnimaliaPseudoscorpionesPseudotyrannochthoniidae

37008F90-45DF-5615-852D-618C7DD10215

https://zoobank.org/AFD5997B-116A-44E4-91B8-F682E488F620

**Figure 5.**
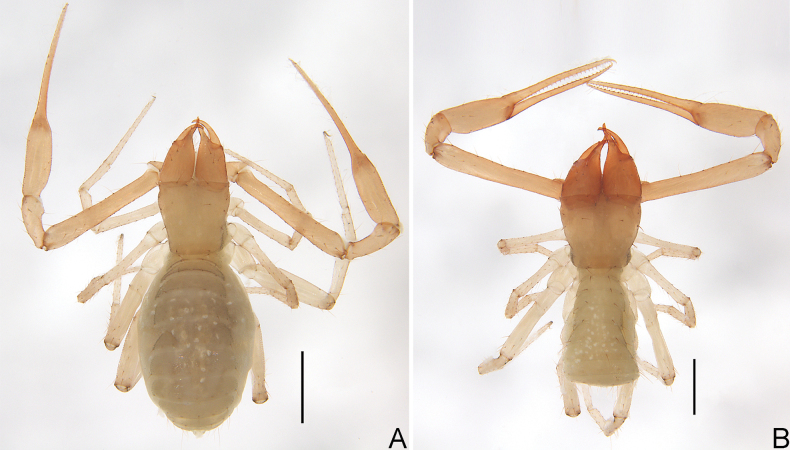
*Spelaeochthoniushuanglaoensis* sp. nov. **A** holotype male, habitus (dorsal view) **B** paratype female, habitus (dorsal view). Scale bars: 0.50 mm.

**Figure 6.**
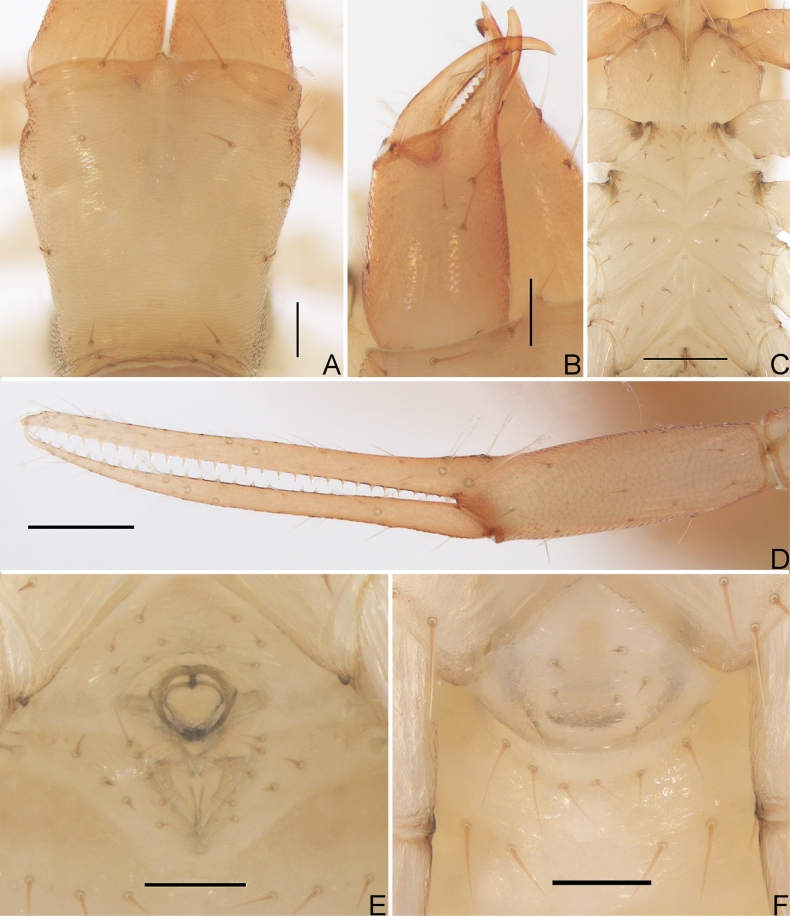
*Spelaeochthoniushuanglaoensis* sp. nov. **A** carapace (dorsal view) **B** left chelicera (dorsal view) **C** coxae (ventral view) **D** left chela (lateral view) **E** male genital area (ventral view) **F** female genital area (ventral view). Scale bars: 0.20 mm (**C, D**); 0.10 mm (**A, B, E, F**).

**Figure 7.**
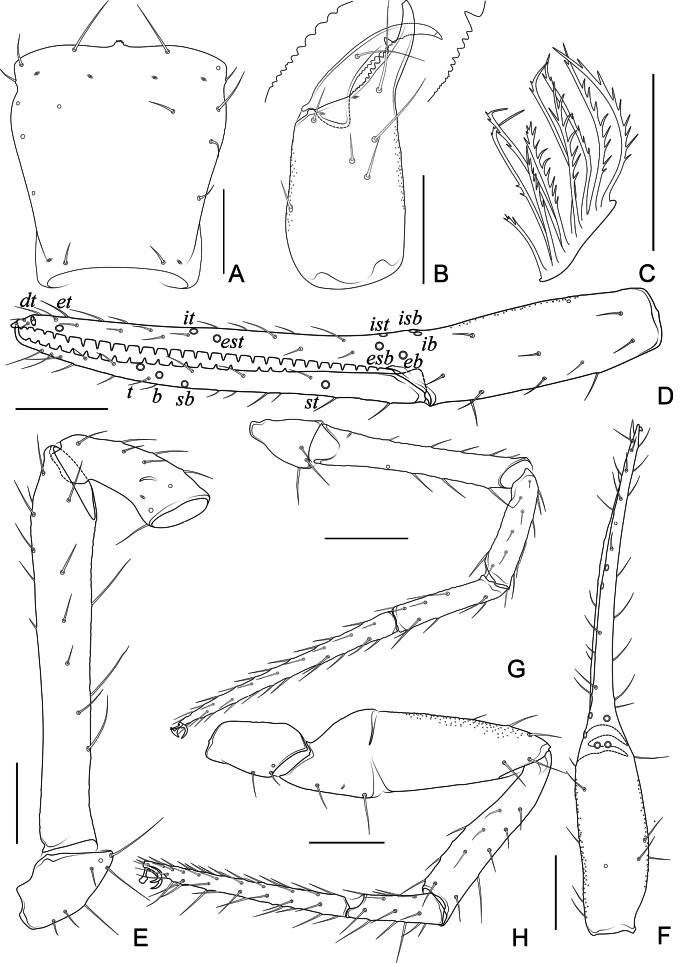
*Spelaeochthoniushuanglaoensis* sp. nov., holotype male **A** carapace (dorsal view) **B** left chelicera (dorsal view), with details of teeth **C** rallum **D** left chela (lateral view), with details of trichobothrial pattern **E** left pedipalp (minus chela, dorsal view) **F** left chela (dorsal view) **G** leg I (lateral view) **H** leg IV (lateral view). Scale bars: 0.20 mm.

**Figure 8.**
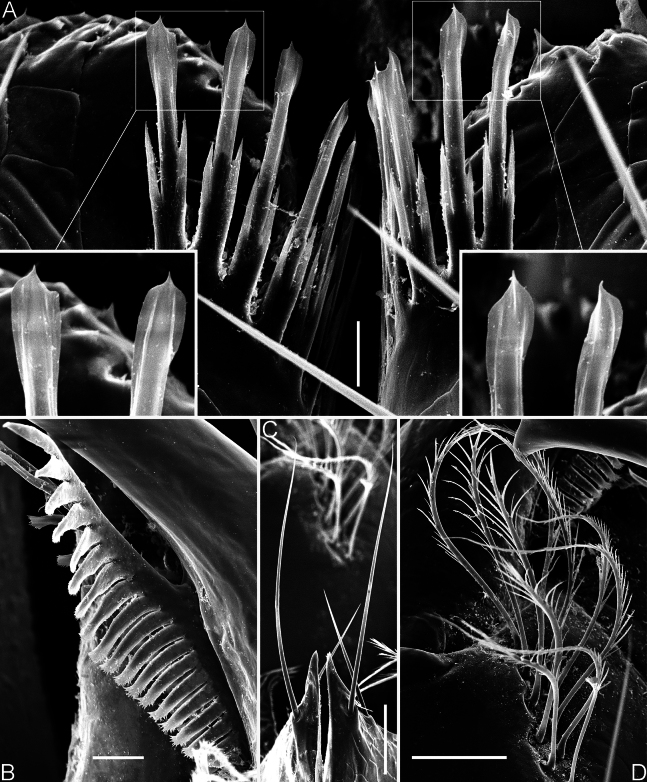
*Spelaeochthoniushuanglaoensis* sp. nov. scanning electron micrographs, paratype female **A** coxal spines in overview, with details of tips **B** serrula exterior **C** manducatory process **D** rallum. Scale bars: 50 μm (**C, D**); 20 μm (**B**); 10 μm (**A**).

Figs 1 , 5 , 6 , 7 , 8

#### Chinese name.

黄老穴伪蝎.

#### Type material.

***Holotype***: China • ♂; Beijing City, Fangshan District, Shidu Town, Wanglaopu Village, Huanglao Cave; 39°40.916′N, 115°39.041′E; 495 m a.s.l.; 19 Oct. 2021; Nana Zhan leg.; under a stone in the deep zone (Fig. 1); Ps.-MHBU-BJFS-21-10-19-02-01. ***Paratype***: • 1♀; same data as for holotype; Ps.-MHBU-BJFS-21-10-19-02-02.

#### Diagnosis

**(♂♀).***Spelaeochthoniushuanglaoensis* sp. nov. is most similar to *S.wulibeiensis* Gao, Hou & Zhang, 2023, but differs from it in having shorter pedipalps (e.g. chela 7.94 (♂), 6.14 (♀) × vs 6.21–6.22 (♂), 5.68 (♀) × longer than broad, length 1.43 (♂), 1.72 (♀) mm vs 1.68–1.74 (♂), 1.76 (♀) mm), 1 additional cheliceral seta (seven vs six), and more numerous fixed chelal finger teeth (29 vs 22–24). It differs from *S.yinae* Li, 2023 in the number of setae on tergite II (four vs two), smaller body size (e.g. chela 7.94 (♂), 6.14 (♀) × vs 5.93 (♂), 6.30 (♀) × longer than broad, length 1.43 (♂), 1.72 (♀) mm vs 1.72 (♂), 1.89 (♀) mm), and more numerous fixed chelal finger teeth (♂) (29 vs 23).

#### Etymology.

The species is named after its type locality, Huanglao Cave.

#### Description.

**Adult male** (Figs 5A, 6A–E, 7). ***Colour***: generally pale yellow; chelicerae, pedipalps and tergites slightly darker; soft parts pale. ***Cephalothorax*** (Figs 6A, C, 7A): carapace inverted-trapezoid, 1.04× longer than broad, gently narrowed posteriorly; surface mostly with fine reticulations; with four anterior lyrifissures and two posterior lyrifissures; no traces of eyes but eye region bulging and convex in dorsal view; epistome present and with some tiny spinules; with 16 setae arranged s4s: 4: 2: 2: 2, most setae heavy, long, and gently curved. Chaetotaxy of coxae: P 3, I 6, II 5, III 4–5, IV 4; manducatory process with two acuminate distal setae, anterior seta less than 1/2 length of medial seta (refer to female, Fig. 8C); coxal spines present on coxa I only, comprising a transverse, contiguous series of six or seven tridentate blades, which arise from a lightly sclerotized or translucent hillock, the central ramus of each blade (except the basal one) sharply acumino-spatulate and extending beyond the lateral rami (refer to female, Fig. 8A); a small, bisetose intercoxal tubercle present between coxae III and IV (Fig. 6C). ***Chelicera*** (Figs 6B, 7B, C): large, approximately as long as carapace, 2.50× longer than broad; six setae and two lyrifissures (exterior condylar lyrifissure and exterior lyrifissure) present on hand, movable finger with one medial seta, all setae acuminate, ventrobasal seta shorter than others. Cheliceral palm with moderate hispid granulation on both ventral and dorsal sides. Both fingers with well-developed teeth, fixed finger with 14 acute teeth, distal one largest; movable finger with 11 retrorse contiguous teeth of equal length; galea absent. Serrula exterior with 19 blades (refer to female, Fig. 8B) and serrula interior with 15 blades. Rallum in two rows and composed of ten finely pinnate blades (11 blades in female), of which the basal-most blade shorter than the others (Figs 7C, 8D). ***Pedipalp*** (Figs 6D, 7D–F): surfaces mostly with fine reticulations; long and slender, trochanter 1.87, femur 6.38, patella 2.69, chela 7.94, hand 3.00× longer than broad; femur 2.37× longer than patella; movable chelal finger 1.69× longer than hand and 0.64× longer than chela. Setae generally long and acuminate; one distal lyrifissure present on patella (Fig. 7E). Chelal palm slightly constricted towards fingers. Fixed chelal finger and hand with eight trichobothria plus duplex trichobothrium (*dt*), movable chelal finger with four trichobothria, *ib*, *isb*, *eb*, *esb*, and *ist* clustered at the base of fixed finger, *esb* slightly distal to *ist*; *it* slightly distal to *est*, situated subdistally and forming a pair; *et* situated subdistally, very close to chelal teeth; *dt* situated distal to *et*, near the tip of fixed finger; *sb* distinctly closer to *b* than to *st* (Fig. 7D). Microsetae (chemosensory setae) absent on hand and both palpal fingers. Sensilla absent. Both chelal fingers with a row of teeth, homodentate, spaced regularly along the margin, larger and well-spaced teeth present in the middle of the row, becoming smaller and closer distally and proximally: fixed chelal finger with 29 teeth, slightly retrorse and pointed; movable chelal finger with 19 teeth (slightly smaller than teeth on fixed chelal finger) (Figs 6D, 7D). Chelal fingers slightly curved in dorsal view (Fig. 7F). ***Opisthosoma***: generally typical, ovate, pleural membrane finely granulated. Tergites and sternites undivided; setae uniseriate and acuminate. Tergal chaetotaxy I–XII: 2: 4: 4: 5: 7: 7: 7: 6: 5: 4: TT: 0. Sternal chaetotaxy III–XII: 9: 8: 10: 9: 10: 9: 7: 8: 0: 2. Anterior genital operculum with nine setae, genital opening pit-like, with seven marginal setae on each side, 23 in total (Fig. 6E). ***Legs*** (Fig. 7G, H): generally typical, long, and slender. Fine granulation present on anterodorsal faces of patella IV. Femur of leg I 1.73× longer than patella and with one lyrifissure at the base of femur; tarsus 2.24× longer than tibia. Femoropatella of leg IV 3.04× longer than deep and with one lyrifissure at the base of femur; tibia 5.80× longer than deep; with a long tactile seta on both tarsal segments: basitarsus 3.86× longer than deep (TS = 0.37), telotarsus 11.40× longer than deep and 2.11× longer than basitarsus (TS = 0.35). Arolium slightly shorter than the claws, not divided; claws simple. ***Dimensions of adult male*** (length/breadth or, in the case of the legs, length/depth in mm). Body length 1.80. Pedipalps: trochanter 0.28/0.15, femur 1.02/0.16, patella 0.43/0.16, chela 1.43/0.18, hand 0.54/0.18, movable finger length 0.91. Chelicera 0.55/0.22, movable finger length 0.28. Carapace 0.53/0.51. Leg I: trochanter 0.21/0.15, femur 0.52/0.09, patella 0.30/0.08, tibia 0.25/0.06, tarsus 0.56/0.05. Leg IV: trochanter 0.27/0.15, femoropatella 0.70/0.23, tibia 0.58/0.10, basitarsus 0.27/0.07, telotarsus 0.57/0.05.

**Adult female** (Figs 5B, 6F, 8). Mostly same as male; tergal chaetotaxy I–XII: 2: 4: 4: 5: 6: 6: 6: 6: 5: 4: TT: 0; sternal chaetotaxy IV–XII: 5: 6: 8: 8: 9: 9: 8: 0: 2; anterior genital operculum with five setae, posterior margin with six marginal setae, 11 in total; leg IV with a long tactile seta on both tarsal segments: basitarsus 3.44× longer than deep (TS = 0.35), telotarsus 9.86× longer than deep and 2.23× longer than basitarsus (TS = 0.36). Body length 1.86. Pedipalps: trochanter 0.35/0.19 (1.84×), femur 1.20/0.20 (6.00×), patella 0.52/0.21 (2.48×), chela 1.72/0.28 (6.14×), hand 0.62/0.28 (2.21×), movable chelal finger length 1.09. Chelicera 0.81/0.33 (2.45×), movable finger length 0.41. Carapace 0.69/0.74 (0.93×). Leg I: trochanter 0.22/0.14 (1.57×), femur 0.56/0.08 (7.00×), patella 0.37/0.08 (4.63×), tibia 0.32/0.07 (4.57×), tarsus 0.68/0.06 (11.33×). Leg IV: trochanter 0.31/0.18 (1.72×), femoropatella 0.82/0.28 (2.93×), tibia 0.68/0.11 (6.18×), basitarsus 0.31/0.09 (3.44×), telotarsus 0.69/0.07 (9.86×).

#### Distribution.

China (Beijing).

### ﻿ Spelaeochthonius tuoliangensis sp. nov.

Taxon classificationAnimaliaPseudoscorpionesPseudotyrannochthoniidae

F72843BC-B441-558B-A92A-2E387CB4C85D

https://zoobank.org/91BA3955-15FC-4214-B45D-2D27571EDCCA

**Figure 9.**
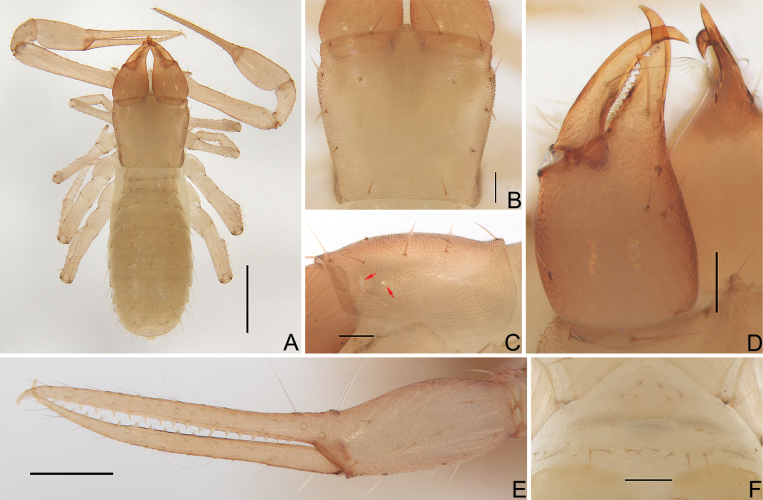
*Spelaeochthoniustuoliangensis* sp. nov., holotype female **A** habitus (dorsal view) **B** carapace (dorsal view) **C** carapace (lateral view), indicate eyespots (red arrows) **D** left chelicera (dorsal view) **E** left chela (lateral view) **F** female genital area (ventral view). Scale bars: 0.50 mm (**A**); 0.20 mm (**E**); 0.10 mm (**B–D, F**).

**Figure 10.**
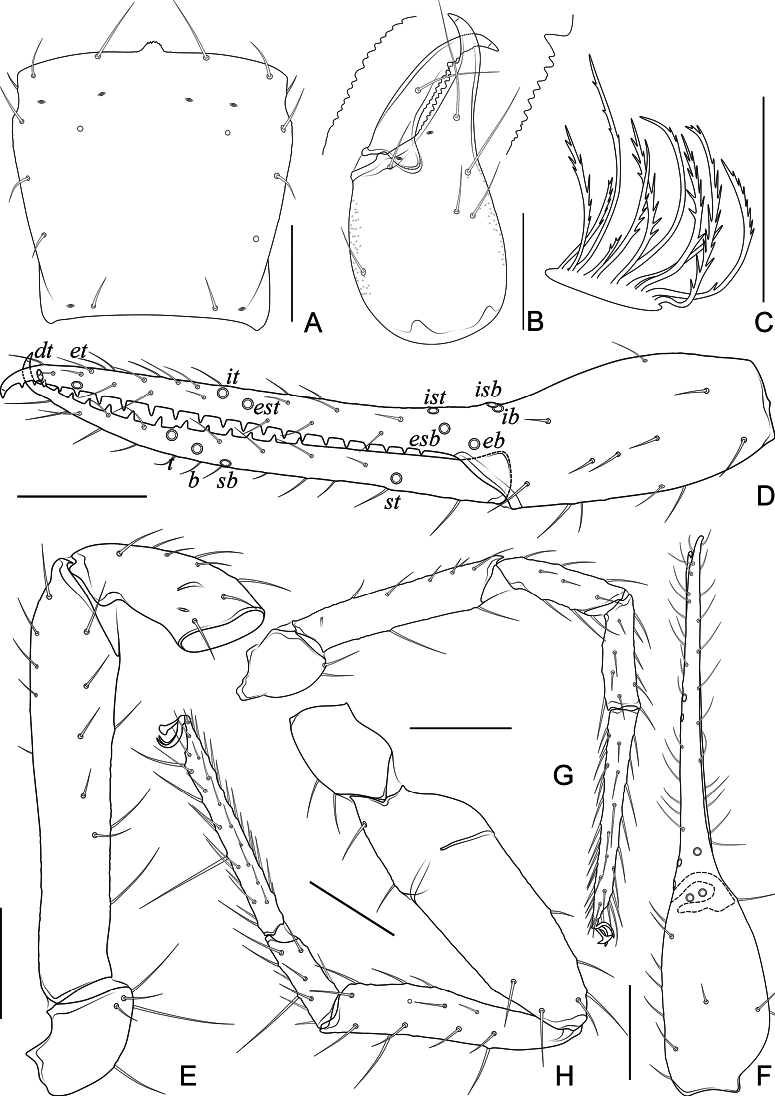
*Spelaeochthoniustuoliangensis* sp. nov., holotype female **A** carapace (dorsal view) **B** left chelicera (dorsal view), with details of teeth **C** rallum **D** left chela (lateral view), with details of trichobothrial pattern **E** left pedipalp (minus chela, dorsal view) **F** left chela (dorsal view) **G** leg I (lateral view) **H** leg IV (lateral view). Scale bars: 0.20 mm.

**Figure 11.**
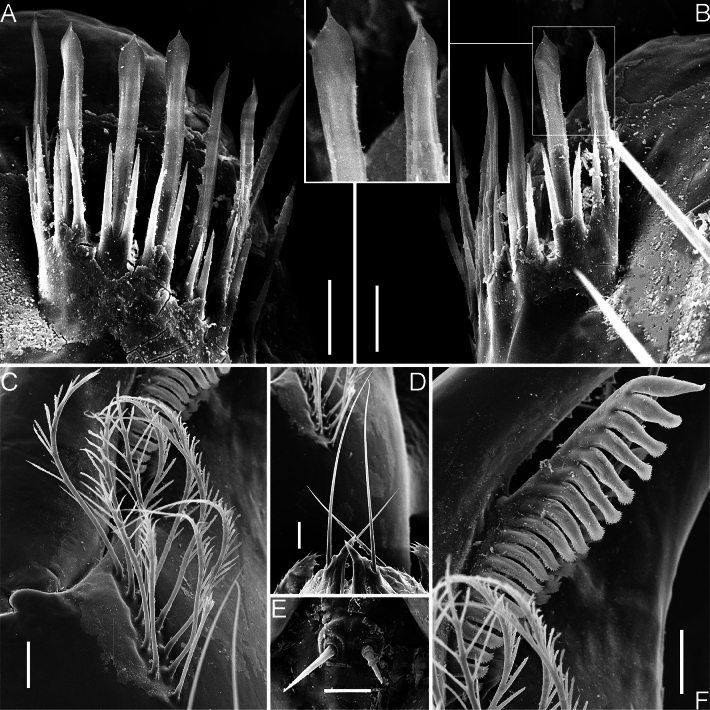
*Spelaeochthoniustuoliangensis* sp. nov. scanning electron micrographs, paratype female **A** left coxal spines **B** right coxal spines, with details of tips **C** rallum **D** manducatory process **E** intercoxal tubercle **F** serrula exterior. Scale bars: 20 μm (**C, D, F**); 10 μm (**A, B, E**).

Figs 1 , 9 , 10 , 11

#### Chinese name.

驼梁穴伪蝎.

#### Type material.

***Holotype***: China • ♀; Hebei Province, Shijiazhuang City, Pingshan County, Tuoliang National Nature Reserve; 38°43.233′N, 113°46.800′E; 1620 m a.s.l.; 13 May. 2018; Xiangbo Guo and Zhaoyi Li leg. (Fig. 1); Ps.-MHBU-HB2018.05.13-01-01. ***Paratype***: • 1♀; same data as for holotype; Ps.-MHBU-HB2018.05.13-01-02.

#### Diagnosis

**(♀).***Spelaeochthoniustuoliangensis* sp. nov. can be separated from its congeners by its visible eyespots. It is most similar to *S.huanglaoensis* sp. nov. but differs from it in having shorter pedipalps (e.g. chela 5.48–5.71× vs 6.14× longer than broad, length 1.15–1.20 mm vs 1.72 mm; palpal femur 5.00–5.13× vs 6.00× longer than broad, length 0.77–0.80 mm vs 1.20 mm) and more setae on tergite I (4 vs 2). It differs from the two congeners from China, *S.wulibeiensis* and *S.yinae*, in having more setae on tergite I (four vs two) and shorter pedipalps (e.g. chela length 1.15–1.20 mm vs 1.76/1.89 mm; palpal femur 5.00–5.13× vs 6.40/7.26× longer than broad, length 0.77–0.80 mm vs 1.28/1.30 mm).

#### Etymology.

This species is named after its type locality, Tuoliang National Nature Reserve.

#### Description.

**Adult females** (male unknown) (Figs 9–11). ***Colour***: generally pale yellow; chelicerae, pedipalps, and tergites slightly darker; soft parts pale. ***Cephalothorax*** (Figs 9B, C, 10A, 11A, B, D, E): carapace inverted-trapezoid, 0.98–1.02× longer than broad, gently narrowed posteriorly; surface mostly with fine reticulations, without furrows but with four anterior lyrifissures and two posterior lyrifissures; with two pairs of eyespots and eye region bulging and convex in dorsal view; epistome present and with some tiny spinules; with 16 setae arranged s4s: 4: 2: 2: 2, most setae heavy, long, and gently curved. Chaetotaxy of coxae: P 3, I 5, II 4, III 4, IV 4; manducatory process with two acuminate distal setae, anterior seta more than 1/2 length of medial seta (Fig. 11D); coxal spines present on coxa I only, comprising a transverse, contiguous series of seven or eight tridentate blades, which arise from a lightly sclerotized or translucent hillock, the central ramus of each blade (except the basal one) sharply acumino-spatulate and extending beyond the lateral rami (Fig. 11A, B); a small, bisetose intercoxal tubercle present between coxae III and IV (Fig. 11E). ***Chelicera*** (Figs 9D, 10B, C, 11C, F): large, approximately as long as carapace, 2.12–2.19× longer than broad; six setae and two lyrifissures (exterior condylar lyrifissure and exterior lyrifissure) present on hand, movable finger with one medial seta, all setae acuminate, ventrobasal seta shorter than others. Cheliceral palm with moderate hispid granulation on both ventral and dorsal sides. Both fingers with well-developed teeth, fixed finger with 12 or 13 acute teeth, distal one largest; movable finger with 14–16 retrorse contiguous teeth of equal length; galea absent. Serrula exterior with 19 blades and serrula interior with 15–17 blades (Fig. 11F). Rallum in two rows and composed of 11 finely pinnate blades (Figs 10C, 11C). ***Pedipalp*** (Figs 9E, 10D–F): surfaces mostly with fine reticulations; long and slender, trochanter 1.53–1.73, femur 5.00–5.13, patella 2.25–2.40, chela 5.48–5.71, hand 2.05–2.10× longer than broad; femur 2.14–2.22× longer than patella; movable chelal finger 1.70–1.77× longer than hand and 0.63–0.65× longer than chela. Setae generally long and acuminate; two distal lyrifissures present on patella (Fig. 10E). Chelal palm slightly constricted towards fingers. Fixed chelal finger and hand with eight trichobothria plus duplex trichobothrium (*dt*), movable chelal finger with four trichobothria, *ib*, *isb*, *eb*, *esb*, and *ist* clustered at the base of fixed finger, *ist* slightly distal to *esb*; *it* slightly distal to *est*, situated subdistally and forming a pair; *et* situated subdistally, very close to chelal teeth; *dt* situated distal to *et*, near the tip of fixed finger; *sb* distinctly closer to *b* than to *st* (Fig. 10D). Microsetae (chemosensory setae) absent on hand and both palpal fingers. Sensilla absent. Both chelal fingers with a row of teeth, homodentate, spaced regularly along the margin, larger and well-spaced teeth present in the middle of the row, becoming smaller and closer distally and proximally: fixed chelal finger with 21 teeth, slightly retrorse and pointed; movable chelal finger with 13 teeth (slightly smaller than teeth on fixed chelal finger) (Figs 9E, 10D). Chelal fingers straight in dorsal view (Fig. 10F). ***Opisthosoma***: generally typical, ovate, pleural membrane finely granulated. Tergites and sternites undivided; setae uniseriate and acuminate. Tergal chaetotaxy I–XII: 4: 5–6: 6: 6: 6: 7: 7: 7: 5–6: 4: TT: 0. Sternal chaetotaxy IV–XII: 12–13: 11–12: 11–12: 9–10: 9–11: 8–9: 8–9: 0: 2. Anterior genital operculum with six setae plus 13 or 14 setae on posterior margin, 19 or 20 in total (Fig. 9F). ***Legs*** (Fig. 10G, H): generally typical, long, and slender. Fine granulation present on anterodorsal faces of femur IV and patella IV. Femur of leg I 1.58–1.71× longer than patella and with one lyrifissure at the base of femur; tarsus 2.09–2.27× longer than tibia. Femoropatella of leg IV 2.76–2.77× longer than deep; tibia 4.90–5.22× longer than deep; with a long tactile seta on both tarsal segments: basitarsus 3.00–3.14× longer than deep (TS = 0.32–0.38), telotarsus 9.20–9.60× longer than deep and 2.09–2.29× longer than basitarsus (TS = 0.35). Arolium slightly shorter than the claws, not divided; claws simple. ***Dimensions of adult females*** (length/breadth or, in the case of the legs, length/depth in mm). Body length 1.71–1.88. Pedipalps: trochanter 0.23–0.26/0.15, femur 0.77–0.80/0.15–0.16, patella 0.336/0.15–0.16, chela 1.15–1.20/0.21, hand 0.43–0.44/0.21, movable finger length 0.73–0.78. Chelicera 0.55–0.57/0.26, movable finger length 0.29. Carapace 0.57/0.56–0.58. Leg I: trochanter 0.16/0.13–0.14, femur 0.41/0.08–0.09, patella 0.24–0.26/0.07, tibia 0.22/0.06, tarsus 0.46–0.50/0.05. Leg IV: trochanter 0.25/0.14–0.15, femoropatella 0.58–0.61/0.21–0.22, tibia 0.47–0.49/0.09–0.10, basitarsus 0.21–0.22/0.07, telotarsus 0.46–0.48/0.05.

#### Distribution.

China (Hebei).

## ﻿Discussion

The morphology of the coxal spines is an important diagnostic feature that allows to distinguish the two Asian endemic genera: *Centrochthonius* and *Spelaeochthonius* (Harvey and Harms 2022; You et al. 2022). In general, *Centrochthonius* shows a unique arrangement of fewer than six coxal blades that are short, tripartite, and distally acute (Gao et al. 2016; Harvey and Harms 2022; Schwarze et al. 2022). In contrast, *Spelaeochthonius* is characterized by having more than seven coxal blades that are longer and distally plumose or terminate as a feathered tassel (Morikawa 1954; You et al. 2022). The two new species of *Spelaeochthonius* described in this study, along with the previously described *S.wulibeiensis*, exhibit typical characters of the genus *Spelaeochthonius*, although with atypical coxal spines that are longer and distally spatulate (Figs 8A, 11A, B; Gao et al. 2023). In addition, the diversity of coxal spine morphology within *Spelaeochthonius* is notable, as seen in *S.undecimclavatus* Morikawa, 1956, where the spines are club-shaped rather than distally plumose (Morikawa 1956). Therefore, it is appropriate to place these two new species in the genus *Spelaeochthonius*, and it may be assumed that the species exhibiting these atypical spines are endemic to China. These atypical spines are similar to those found in the three North American species classified as “*Pseudotyrannochthonius*” and forming a monophyletic sister group to *Spelaeochthonius* (Harms et al. 2024); these are all characterized by tripartite spines with spatulate tips. However, the intermediate rami of these atypical spines are notably elongated (Muchmore 1967; Benedict and Malcolm 1970).

All 11 currently known *Spelaeochthonius* species are exclusively found within caves and are completely blind (WPC 2024). *Spelaeochthoniustuoliangensis* sp. nov. represents the first epigean species of this genus with small eye spots (Fig. 9C). While most China’s karst landforms are distributed in the southern subtropical regions, there are also a few karst caves located in temperate regions (Liu et al. 2020). Due to Pleistocene glaciation, caves served as refugia for troglobites, like *S.huanglaoensis* sp. nov., while their surface counterparts would have normally gone extinct under adverse climatic conditions (Holsinger 2000). *Spelaeochthoniustuoliangensis* sp. nov. may be the remnant of a former surface fauna of *Spelaeochthonius* that is now largely extinct in eastern Asia but remains highly diverse in subterranean habitats. The discovery of two new *Spelaeochthonius* species further extends the geographic range of the genus in East Asia. In contrast, the genus *Allochthonius*, which is also endemic to East Asia, is more widely distributed; the discovery of *Allochthonius* in Baltic amber from Europe (Schwarze et al. 2022) indicates a previously wider distribution of this genus. The larger population and perhaps greater adaptability of *Allochthonius* have allowed this genus to occupy a wider range of ecological niches.

The research on the family Pseudotyrannochthoniidae is still in its infancy in China, with 21 species recorded thus far (Fig. 1; WPC 2024), mostly concentrated in Yunnan and Guizhou provinces of southwestern China. More investigations are needed in northern and central China to explore the geographic range of this family. However, our fieldwork has revealed that these small arachnids have very low abundance, are endemic to small areas (some are confined to a single cave), are vulnerable to environmental changes, and are easily overlooked. Therefore, it is extremely important to protect their habitat while investigating.

## Supplementary Material

XML Treatment for Allochthonius

XML Treatment for Allochthonius hispidus

XML Treatment for Spelaeochthonius

XML Treatment for Spelaeochthonius huanglaoensis

XML Treatment for Spelaeochthonius tuoliangensis

## ﻿Additional information

### Author ORCIDs

Yanmeng Hou https://orcid.org/0000-0003-0059-3419

Feng Zhang https://orcid.org/0000-0002-3347-1031

### Data availability

All of the data that support the findings of this study are available in the main text.

## References

[B1] Beier M (1932) Pseudoscorpionidea I. Subord. Chthoniinea et Neobisiinea. Tierreich 57: [i–xx,] 1–258. 10.1515/9783111435107

[B2] Benedict EM Malcolm DR (1970) Some pseudotyrannochthoniine false scorpions from western North America (Chelonethida: Chthoniidae). Journal of the New York Entomological Society 78: 38–51.

[B3] Chamberlin JC (1929) On some false scorpions of the suborder Heterosphyronida (Arachnida - Chelonethida). Canadian Entomologist 61(7): 152–155. 10.4039/Ent61152-7

[B4] Chamberlin JC (1931) The arachnid order Chelonethida. Stanford University Publications, University Series (Biological Science) 7(1): 1–284.

[B5] Gao ZZ Zhang YF Zhang F (2016) Two new species of Pseudotyrannochthoniidae, including the first species of *Pseudotyrannochthonius* (Pseudoscorpiones) from China. Acta Zoologica Academiae Scientiarum Hungaricae 62(2): 117–131. 10.17109/AZH.62.2.117.2016

[B6] Gao ZZ Hou YM Zhang F (2023) Four new species of cave-adapted pseudoscorpions (Pseudoscorpiones, Pseudotyrannochthoniidae) from Guizhou, China. ZooKeys 1139: 33–69. 10.3897/zookeys.1139.96639 36761277 PMC9843613

[B7] Harms D (2018) The origins of diversity in ancient landscapes: Deep phylogeographic structuring in a pseudoscorpion (Pseudotyrannochthoniidae: *Pseudotyrannochthonius*) reflects Plio-Pleistocene climate fluctuations. Zoologischer Anzeiger 273: 112123. 10.1016/j.jcz.2018.01.001

[B8] Harms D Harvey MS (2013) Review of the cave-dwelling species of *Pseudotyrannochthonius* Beier (Arachnida: Pseudoscorpiones: Pseudotyrannochthoniidae) from mainland Australia, with description of two troglobitic species. Australian Journal of Entomology 52(2): 129–143. 10.1111/aen.12009

[B9] Harms D Roberts JD Harvey MS (2019) Climate variability impacts on diversification processes in a biodiversity hotspot: A phylogeography of ancient pseudoscorpions in south-Western Australia. Zoological Journal of the Linnean Society 186(4): 934–949. 10.1093/zoolinnean/zlz010

[B10] Harms D Harvey MS Roberts JD Loria SF (2024) Tectonically driven climate change and the spread of temperate biomes: Insights from dragon pseudoscorpions (Pseudotyrannochthoniidae), a globally distributed arachnid lineage. Journal of Biogeography 51(6): 1032–1048. 10.1111/jbi.14801

[B11] Harvey MS (1992) The phylogeny and classification of the Pseudoscorpionida (Chelicerata: Arachnida). Invertebrate Systematics 6(6): 1373–1435. 10.1071/IT9921373

[B12] Harvey MS Harms D (2022) The pseudoscorpion genus *Centrochthonius* (Pseudoscorpiones: Pseudotyrannochthoniidae) from central Asia and description of a new species from Nepal. The Journal of Arachnology 50(2): 158–174. 10.1636/JoA-S-21-033

[B13] Holsinger JR (2000) Ecological derivation, colonization, and speciation. In: Wilkens H Culver DC Humphries WF (Eds) Subterranean Ecosystems.Elsevier, Amsterdam, 399–415.

[B14] Hu JF Zhang F (2012) Two new species of the genus *Allochthonius* Chamberlin from China (Pseudoscorpiones: Pseudotyrannochthoniidae). Entomologica Fennica 22(4): 243–248. 10.33338/ef.5003

[B15] Judson MLI (2007) A new and endangered species of the pseudoscorpion genus *Lagynochthonius* from a cave in Vietnam, with notes on chelal morphology and the composition of the Tyrannochthoniini (Arachnida, Chelonethi, Chthoniidae). Zootaxa 1627(1): 53–68. 10.11646/zootaxa.1627.1.4

[B16] Liu K Sun WJ Wang SS Sun Y (2020) Study on the characteristics of karst development in Beijing. Carbonates and Evaporites 35(2): 1–13. 10.1007/s13146-020-00584-7

[B17] Morikawa K (1954) On some pseudoscorpions in Japanese lime-grottoes. Memoirs of Ehime University 2(2B): 79–87.

[B18] Morikawa K (1956) Cave pseudoscorpions of Japan (I). Memoirs of Ehime University 2(2B): 271–282.

[B19] Morikawa K (1960) Systematics studies of Japanese pseudoscorpions. Memoirs of Ehime University 2(2B): 85–172.

[B20] Muchmore WB (1967) Pseudotyrannochthoniine pseudoscorpions from the western United States. Transactions of the American Microscopical Society 86(2): 132–139. 10.2307/3224679

[B21] Sakayori H (2000) A new species of the genus *Allochthonius* (Pseudoscorpion, Chthoniidae) from Mt. Kohshin, Tochigi Prefecture, Central Japan. Edaphologia 65: 13–18.

[B22] Schwarze D Harms D Hammel J Kotthoff U (2022) The first fossils of the most basal pseudoscorpion family (Arachnida: Pseudoscorpiones: Pseudotyrannochthoniidae): evidence for major biogeographical shifts in the European paleofauna. Palaontologische Zeitschrift 96(1): 11–27. 10.1007/s12542-021-00565-8

[B23] Viana ACM Ferreira RL (2021) A new troglobitic species of Allochthonius (subgenus Urochthonius) (Pseudoscorpiones, Pseudotyrannochthoniidae) from Japan. Subterranean Biology 37: 47–55. 10.3897/subtbiol.37.58580

[B24] World Pseudoscorpiones Catalog (2024) World Pseudoscorpiones Catalog. Natural History Museum Bern. https://wac.nmbe.ch/order/pseudoscorpiones/3 [Accessed on 16.04.2024]

[B25] You JY Yoo JS Harvey MS Harms D (2022) Some cryptic Korean karst creatures: revalidation of the pseudoscorpion genus *Spelaeochthonius* (Pseudoscorpiones: Pseudotyrannochthoniidae) and description of two new species from Korea. The Journal of Arachnology 50(2): 135–157. 10.1636/JoA-S-21-025

[B26] Zhang FB Zhang F (2014) A new species of the genus *Allochthonius* (Pseudoscorpiones, Pseudotyrannochthoniidae) from Liupan Mountains, China, with the description of the male of *Allochthoniusbrevitus*. Acta Zoologica Academiae Scientiarum Hungaricae 60: 45–56.

